# Social Media Listening in Congenital Ichthyosis: Quantitative and Qualitative Findings

**DOI:** 10.2196/79761

**Published:** 2026-03-18

**Authors:** Maëlla Severino-Freire, Céline Granier Tournier, Joëlle Malaab, Kira Süßmuth, Antoni Gostynski, Ángela Hernández-Martín, Juliette Mazereeuw-Hautier

**Affiliations:** 1 Dermatology Department, Reference Center for Rare Skin Diseases, Centre Hospitalier Universitaire de Toulouse, 24 chemin de Pouvourville, Toulouse, 31059, France, 33 0 33 5 67 77 8; 2 Kap Code, Paris, France; 3 Department of Dermatology and Allergology, Campus of Medical School Berlin, Helios Klinikum Berlin-Buch, Berlin, Germany; 4 Dermatology Department, Medical Center, Maastricht University, Maastricht, The Netherlands; 5 Dermatology Department, Hospital Infantil Universitario Niño Jesús, Madrid, Spain; 6 Toulouse University, CNRS, Inserm, Institut Toulousain des Maladies Infectieuses et Inflammatoires, Toulouse, France

**Keywords:** social media, congenital ichthyosis, quality of life, unmet needs

## Abstract

This study analyzed social media posts from patients with congenital ichthyosis and their caregivers across Europe and found that users, primarily young women, discussed hygiene care, psychological impact, therapeutic challenges, and lack of disease awareness. This allowed for the identification of unmet needs and potential actions to improve patients’ quality of life.

## Introduction

Congenital ichthyoses (CI), recently classified within the group of epidermal differentiation disorders, are chronic skin conditions characterized by anomalies such as scaling or skin thickening, often associated with erythroderma [1]. Quality of life is often impaired because of distressing symptoms, limited treatment efficacy, demanding skin care routines, social stigmatization, and a lack of awareness or education among both health care professionals and the general public [2,3]. Consequently, patients often turn to the internet to find answers, connect with other patients, seek support, and access information. In this study, we analyzed the role of the internet in the lives of patients with CI to better understand their experiences and needs, with the goal of improving patient care and management.

## Methods

### Study Design and Population

This observational, retrospective, real-world study used data collected from social media posts by patients with CI and their caregivers. The messages, written in the local languages, were geolocated in four European countries: France, Germany, the Netherlands, and Spain. This study covered the period from January 2014 through January 2024.

### Data Extraction and Analysis

Data extraction and analysis methods are detailed in Multimedia Appendix 1. Briefly, data extraction was performed using the digital consumer intelligence platform Brandwatch to collect publicly available posts from public platforms and health-related forums (listed in Multimedia Appendix 2) using a list of CI-related keywords defined by the investigators (listed in Multimedia Appendix 3). Posts were cleaned by removing duplicates, irrelevant sources, nontarget languages, and messages that were too short or excessively long. A supervised machine-learning pipeline based on 2 sequential XGBoost classifiers was then applied to identify patient and caregiver experiences. Data analysis included manual extraction of demographic information when explicitly mentioned and identification of the main discussion themes using unsupervised topic modeling (biterm topic modeling), followed by human interpretation.

### Ethical Considerations

This study used data exclusively from publicly accessible sources, thereby excluding private groups and web pages. Ethical approval was not required, as users implicitly consented to the reuse of their data by posting on public platforms, and all the results were presented without identifiable information. Sensitive details, including names, usernames or handles, geographic locations, and other personal data, were deleted. To ensure compliance with the General Data Protection Regulation, the names of treatments were anonymized and replaced with a generic [TREATMENT] placeholder in the messages.

## Results

A total of 5175 messages were initially collected. After removing off-topic posts, 4302 messages from 2182 unique users remained. Following the application of the patient and caregiver classification algorithm, 201 messages from 169 users were identified. Of these users, 90 were patients and 79 were caregivers.

The characteristics of the users and their posts are reported in Table 1. Of the 169 users, 110 (65%) were women, with a mean age of 36 years (SD 6.1; range 21‐57). Among the 43 identified social media networks (Multimedia Appendix 2), the most frequently used platforms were Twitter (31.3%, 63/201), Doctissimo (18.4%, 25/201), and Facebook (7.5%, 15/201). The 5 main discussion topics, in decreasing order of frequency, were hygiene care (31%, 63/201), psychological impact of the disease (18%, 37/201), disease characteristics (17%, 35/201), contact tracing (13%, 27/201), and treatments or lack of awareness of the disease (10%, 20/201). Content and examples of posts for each topic are provided in Table 2.

**Table 1.** Characteristics of the users (N=169) and posts (N=201).

Characteristics	Values
**Demographic data of the users, n (%)**	
Users	
Patients	90 (53.3)
Caregivers	79 (46.7)
Gender	
Female	110 (65.1)
Male	27 (16)
Not mentioned	32 (18.9)
Age (y)	
18-19	0 (0)
20-29	27 (16)
30-39	105 (62.1)
40-49	30 (17.8)
50-59	3 (1.8)
60 and older	0 (0)
Not mentioned	4 (2.4)
Mean age (SD); range (y)	36 (6.1); 21‐57
**Characteristics of the posts, n (%)**	
Topics of discussion	
Hygiene care	63 (31.3)
Psychological impact	37 (18.4)
Characteristics of the disease	35 (17.4)
Request for contact and discussions with ichthyosis patients or families	27 (13.4)
Information on available treatments and feedback	20 (10.0)
Other^a^	19 (9.5)
Social media platforms	
Twitter	63 (31.3)
Doctissimo	25 (12.4)
Facebook	15 (7.5)
Other^b^	98 (48.8)
Countries	
Germany	76 (37.8)
France	58 (28.9)
Spain	54 (26.9)
The Netherlands	13 (6.5)

a Includes doubts about the diagnosis and questions from parents about their child’s case.

b The complete list of social media platforms analyzed is presented in Multimedia Appendix 2.

**Table 2.** Description of the 5 main discussion topics and examples of posts

Topics of discussion	Examples of posts
**Hygiene care**	
Description of their skin conditions and the type of ichthyosis they have. Which skincare products (creams, lotions, soaps) improve their skin. Their routines based on personal experience or dermatologist recommendations. Experience with different products. Seasonal influence.	*I may have a solution that can work..., it depends on the type of ichthyosis you have. There’s a cream that could help you,....* *Our son suffers from ichthyosis. In winter, the skin is in really bad shape, but in summer we have it under control.*
**Psychological impact**	
People’s perception was a major concern of patients. Patients felt burdened by how others looked at them outside their family circle. Patients often had to explain their visibly different skin; this was especially difficult for adolescents and young adults, who struggled with feeling “different.” Caregivers, particularly parents, were also affected by others’ reactions and questions. In some cases, these encounters felt like harassment and created a strong sense of injustice.	*I’ve heard advice and comments all my life about my ichthyosis and I’m just sick of it.* *Have you ever been reported to social services for lack of knowledge of this disease, on the pretext that your children are dirty? In my case, I have 2 children with ichthyosis and I feel harassed and tired of fighting against a steel wall...*
**Characteristics of the disease**	
Physical burden was a key theme across many posts. Patients and caregivers described skin lesion location, appearance, and symptoms, and functional issues such as the inability to perspire or difficulty regulating body temperature. In some cases, skin lesions were highly visible and disruptive to daily life. Caregivers often expressed a sense of helplessness in managing these symptoms.	*My skin is dying with the cold + humidity and I’m in agony. Damn Ichthyosis.* *I was scratching and bleeding because the itching was so much worse than anything else.*
**Contact tracing**	
Need for connection and information. Seeking advice from others due to unpreparedness of dealing with child’s condition. Dissatisfaction with medical advice led them to prefer peer feedback. Created blogs to share experiences, bridge the information gap, and simply be heard. Seeking emotional support from persons who understand and do not judge.	*Hello, after much research we have been diagnosed with ichthyosis in our three-month-old adopted baby. Now, there are a lot of questions in the room, does anyone have any experience in this area?* *I am the mother of a little boy who is 10 1/2 months old. He has Netherton syndrome and was hospitalized until 8 1/2 months....I would like to hear from parents who know about this disease and who can tell me about their daily lives..., parents who could give me advice, etc....to help my baby. Thank you to everyone.*
**Treatments and lack of awareness of the disease**	
Through their experiences—both positive and negative—patients and caregivers shared opinions and reported on various treatments they have tried or been offered. Many highlighted the general lack of awareness about the disease among those around them. This lack of understanding was reported as a challenge in daily life, including interactions with family, friends, schools, and nurseries. The main issue, from the patient and caregiver perspective, is the limited recognition of the condition by some health care professionals, which can strain the relationships between patients, caregivers, and doctors.	*Hello my child has been suffering from second degree ichthyosis since birth and the only thing that is always suggested is cortisone....side effects are severe and unacceptable...but there is virtually no alternative for my child.* *When my son went to nursery, one of the nursery nurses thought he had spots and tried to rub his legs. When she told me, I explained to her that it was ichthyosis and she had never heard of it.*

## Discussion

This first European study on social media posts from users with CI and their caregivers found that users, predominantly young women on Twitter, primarily discussed hygiene care, therapeutic issues, psychological impact, and low disease awareness among the public and health care professionals.

Regarding the study population, we observed a predominance of young female users, consistent with the general tendency for women to seek health information online [4], and with the findings of Voillot et al [5] in atopic dermatitis (AD), suggesting both general and disease-specific trends.

Hygiene care and therapeutic issues were also key discussion topics for patients with AD, along with peer support [5]. In contrast, lack of disease awareness was specific to CI, a rare condition, and, together with the psychological impact, had already been highlighted in our 2012 focus group study [2]. Topics such as romantic relationships and sexual health, reported by van Veen [3] and a shared patient voices report [6], were absent in our study, either because patients did not raise them or because they were removed as sensitive information.

Inadequate management, linked to gaps in health care professionals’ knowledge or interest and limited effective therapies, was expressed more strongly online, possibly due to the anonymity of social media. Unlike the 2012 qualitative study [2], no positive aspects of living with CI were reported, suggesting that social media mainly serves as a space to voice challenges rather than share positive experiences.

This study has several limitations, including the small number of posts due to the rarity of the disease, the exclusion of data from private groups and Instagram (Multimedia Appendix 1), and the possibility that it did not capture the needs and experiences of all patients, as those uncomfortable with the internet may not have participated.

In conclusion, this study highlights the needs of patients with CI that may guide improved management. Providing information, particularly through online tools developed in collaboration with patient associations, rare disease networks [7], or digital education programs such as our e-TPE (digital therapeutic patient education) is essential [8]. Public education also remains important and could be promoted through national society campaigns. The growing use of artificial intelligence, as recently explored in AD, is likely to transform how patients seek information [8-10]. Psychological support should be routinely offered to patients and caregivers, and research should continue to focus on developing new therapies. Collectively, these measures can help enhance patients’ quality of life.

## Supplementary material

10.2196/79761 Multimedia Appendix 1 Supplementary methods.

10.2196/79761 Multimedia Appendix 2 Social media used in the different countries.

10.2196/79761 Multimedia Appendix 3 Query used for extraction on Brandwatch.

## Abbreviations

AD: atopic dermatitis
CI: congenital ichthyosis

## References

[1] Hernández-Martín Á, Paller AS, Sprecher E (2025). A proposal for a new pathogenesis-guided classification for inherited epidermal differentiation disorders. Br J Dermatol.

[2] Mazereeuw-Hautier J, Dreyfus I, Barbarot S (2012). Factors influencing quality of life in patients with inherited ichthyosis: a qualitative study in adults using focus groups. Br J Dermatol.

[3] van Veen FCAP, Rossel V, Steijlen PM (2025). The perceived quality of life in adult patients with inherited ichthyosis: a qualitative interview study. Br J Dermatol.

[4] Fox S (2011). Profiles of health information seekers. Pew Research Center.

[5] Voillot P, Riche B, Portafax M (2022). Social media platforms listening study on atopic dermatitis: quantitative and qualitative findings. J Med Internet Res.

[6] van Veen FCAP, Sprecher E, Schwartz J (2025). The importance of shared patient voices in advancing science for genodermatoses on a global scale. J Invest Dermatol.

[7] Documents for patients. ERN-Skin (European Reference Networks for Rare Skin Diseases).

[8] Sulejmani P, Negris O, Aoki V (2024). A large language model artificial intelligence for patient queries in atopic dermatitis. J Eur Acad Dermatol Venereol.

[9] Ayers JW, Poliak A, Dredze M (2023). Comparing physician and artificial intelligence chatbot responses to patient questions posted to a public social media forum. JAMA Intern Med.

[10] Sulejmani P, Negris O, Aoki V (2024). The global reach of artificial intelligence in atopic dermatitis: the quality and reliability of ChatGPT responses in 8 languages. JAAD Int.

