# Supplementary material for: Social Media Listening in Congenital Ichthyosis: Quantitative and Qualitative Findings
Source: JMIR Form Res. 2026 Mar 18;10:e79761. doi: 10.2196/79761 (PMC12998599; doi:10.2196/79761)
Supplement: Multimedia Appendix 1 [file formative-v10-e79761-s001.docx]

**Multimedia Appendix 1: Supplementary methods**

**Data extraction**

The dataset comprised messages obtained from public platforms such as X (formerly Twitter) and health-related forums including Doctissimo and rund-ums-baby.de. Due to restricted data access, the analysis was limited to public Facebook pages and open groups, while Instagram and WhatsApp were excluded due to the complexity of their data extraction via Application Programming Interfaces (APIs). A detailed list of ichthyosis-related keywords (e.g., ichthyosis, Netherton, harlequin) was meticulously compiled in the respective languages of each country. To enhance exhaustiveness and account for potential misspellings, multiple variations of the keywords were included. This comprehensive list of keywords was utilized in the data extraction query (Multimedia Appendix 3).

Data extraction was performed by the Brandwatch® extractor (Cision Ltd.) ^1^. Publicly available posts were collected from Twitter and forums containing one of the relevant keywords. Simultaneously, a web crawling process gathered information from publicly accessible Facebook pages. Posts were retrieved along with their associated metadata (e.g., author, publication date), resulting in a dataset created in Microsoft Excel.

To ensure uniform consideration and analysis, no distinction was made between posts from different platforms. During the pre-processing phase, relevant messages were selected based on specific exclusion criteria. Posts containing five words or fewer, as well as those exceeding 10,000 characters, were excluded due to their lack of relevance. Duplicates and sources deemed unreliable or irrelevant to the study (e.g., advertising websites, forums about cars, pets, or animals) were also removed. Additionally, posts not written in French, German, Dutch, or Spanish were excluded from the analysis.

To further refine the filtering process, a supervised machine learning algorithm was employed to identify posts related to patients’ or caregivers’ experiences. This algorithm was previously developed using a training set comprising 12,330 messages from various health domains, including dermatology, tobacco use, and oncology. The methodology involves a pipeline featuring two sequential XGBoost ^2^ classifiers: one for identifying caregivers’ experiences and the other for identifying patients’ experiences. This approach allowed for the classification of posts as belonging to patients, caregivers, or neither. The classifiers leverage features combining pronouns and lexical fields related to relatives and pathologies (e.g., "my [pronoun] father [relative] has cancer [pathology]"). Initially, the algorithm was trained to identify caregiver-related messages across the entire dataset. Subsequently, it was reapplied to the remaining dataset, excluding the already identified caregiver messages, to identify patients' messages. Performance evaluation of the classifiers yielded F1 scores, which combine precision and recall, of 88% for the caregiver classifier and 87% for the patient classifier.

In the present study, only posts from patients and caregivers were considered for analysis.

**Data Analysis: Demographics and Topics of discussion**

The age and gender of patients or caregivers was determined through manual examination of the text, particularly when this information was explicitly provided, as illustrated by the following example:"[...] my mother was diagnosed with bladder cancer at the age of 71."

In instances where age and gender information were not explicitly mentioned, the data was categorized as "undetermined."

Main discussion themes were identified through the examination of all posts from patients regarding ichthyosis, utilizing Biterm Topic Modeling through the BTM R package ^3^. BTM is a natural language processing and text mining technique that clusters similar texts based on common discussion topics and generates lists of words for cluster labelling ^4^. In topic modeling, documents (messages/posts) are considered as mixtures of topics, which are probability distributions over the words in the dataset. Consequently, a post can be associated with its most prominent topic. BTM provides, for each topic, a list of the twenty highest probability words along with all the posts associated with that topic. To ensure an unbiased analysis, BTM was applied without any presuppositions about the topics that might emerge from the data. This allowed the algorithm to organically group texts into categories based solely on the statistical significance of word patterns, free from human preconceptions. As a result, BTM automatically organized the posts into categories, ranked by their frequency of appearance in the dataset. Each category was characterized by a list of its most frequently occurring words, serving as a fingerprint for each topic. These word lists enabled the identification of the central theme of the discussions within each category. Through human interpretation, these lists of words were used to label the topics, and the associated posts were thoroughly examined to ensure accurate interpretation.

**References**

1. Brandwatch Consumer Research [Internet]. Brandwatch. [cited 2022 Jul 25]. Available from: https://www.brandwatch.com/products/consumer-research/features/

2. Chen T, He T, Benesty M, Khotilovich V, Tang Y, Cho H, et al. Xgboost: extreme gradient boosting. R package version 04-2. 2015;1(4):1–4.

3. Yan X. shortext.github.io [Internet]. 2021 [cited 2022 Aug 11]. Available from: https://github.com/xiaohuiyan/xiaohuiyan.github.io/blob/61d74f1b74a26ba136d65c7b3420d1e54440fe44/paper/BTM-WWW13.pdf

4. Yan X, Guo J, Lan Y, Cheng X. A biterm topic model for short texts. In: Proceedings of the 22nd international conference on World Wide Web [Internet]. New York, NY, USA: Association for Computing Machinery; 2013 [cited 2024 Sep 17]. p. 1445–56. (WWW ’13). Available from: https://doi.org/10.1145/2488388.2488514
