# Supplementary material for: Social Media Listening in Congenital Ichthyosis: Quantitative and Qualitative Findings
Source: JMIR Form Res. 2026 Mar 18;10:e79761. doi: 10.2196/79761 (PMC12998599; doi:10.2196/79761)
Supplement: Multimedia Appendix 2 [file formative-v10-e79761-s002.docx]

**Multimedia Appendix 2: Used social media in the different countries**

| **SOURCES (LANGUAGES)** | **NUMBER OF MESSAGES/ NUMBER OF INTERNET USERS** |
| --- | --- |
| TWITTER (G/SP/FR/D) | 63/42 |
| DOCTISSIMO (FR) | 25/24 |
| FACEBOOK (SP/FR) | 15/15 |
| FOROCOCHES.COM (SP) | 6/6 |
| RUND-UMS-BABY.DE (G) | 6/5 |
| URBIA.DE (G) | 6/6 |
| BABYCENTER.COM (SP) | 5/2 |
| GOFEMININ.DE (G) | 5/4 |
| REHAKIDS.DE (G) | 5/4 |
| SWISSMOMFORUM.CH (G) | 5/4 |
| BABYCENTER.DE (G) | 4/4 |
| GUTEFRAGE.NET (G) | 4/4 |
| MAMACOMMUNITY.DE (G) | 4/2 |
| BABYCENTER.FR (FR) | 3/2 |
| PSORIASIS-NETZ.DE (G) | 3/3 |
| RHEUMA-ONLINE.DE (G) | 3/3 |
| WUNSCHKINDER.NET (G) | 3/3 |
| babyforum.de (G) | 2/2 |
| chefkoch.de (G) | 2/2 |
| dosfarma.com (SP) | 2/2 |
| fok.nl (D) | 2/2 |
| ht-mb.de (G) | 2/2 |
| mendrulandia.es (SP) | 2/2 |
| mforos.com (SP) | 2/2 |
| 24baby.nl (D) | 2/2 |
| beauté test (FR) | 1/1 |
| carenity.com (SP) | 1/1 |
| carenity.de (G) | 1/1 |
| crianzanatural.com (SP) | 1/1 |
| dokter.nl (D) | 1/1 |
| elpais.com (SP) | 2/2 |
| hipp.de (G) | 1/1 |
| iriteser.de (G) | 1/1 |
| jeux video (FR) | 1/1 |
| journaldesfemmes.com (FR) | 1/1 |
| kleiderkreisel.de (G) | 1/1 |
| med1.de (G) | 1/1 |
| nemokennislink.nl (D) | 1/1 |
| neurodermitisportal. de (G) | 2/2 |
| onmeda.de (G) | 1/1 |
| rezepturforum.de (G) | 1/1 |
| schnullerfamilie.de (G) | 1/1 |
| zwangerschapspagina.nl (D) | 1/1 |
| TOTAL | 201/169 |

Abbreviations : D : Dutch ; FR : French ; G : German ; SP : Spanish
