# Supplementary material for: Social Media Listening in Congenital Ichthyosis: Quantitative and Qualitative Findings
Source: JMIR Form Res. 2026 Mar 18;10:e79761. doi: 10.2196/79761 (PMC12998599; doi:10.2196/79761)
Supplement: Multimedia Appendix 3 [file formative-v10-e79761-s003.docx]

**Multimedia Appendix 3: Query used for extraction on Brandwatch**

**France:** ichtyose OR Icthyose OR ictyose OR Ichthyosis OR ictiose OR ichtyosique OR ichtyosiforme OR ichthyosiform OR ichtyosic OR trichothiodystrophie OR trichothiodystrophy OR "loricrin keratoderma" OR "erythrokeratodermia variabilis" OR "érythrokératodermie variable" OR "Multiple sulfatase deficiency" OR "Déficit multiple en sulfatase" OR "Ichthyosis-hypotrichosis-sclerosing" OR "Ichthyose-hypotrichose-sclérosante" OR "Ichthyosis-short"

OR ((bébé OR enfant OR baby) NEAR/5 (collodion OR Arlequin OR Harlequin))

OR (("syndrome" OR "sindrome" OR "syndrom") NEAR/3 ((Gaucher NEAR/3 "type 2") OR "Netherton" OR "Harlequin" OR "Arlequin" OR "Sjögren Larsson" OR "Sjögren-Larsson" OR "KID" OR "SAM" OR "Refsum" OR "peeling skin" OR "peeling-skin" OR "ARCI" OR "KLICK" OR "IFAP" OR "Conradi-Hünermann-Happle" OR "Conradi Hünermann Happle" OR "TDD" OR "MEDNIK" OR "CEDNIK" OR "Neu-Laxova" OR "Neu Laxova" OR "CHIME" OR "SAM" OR "HELIX" OR "Sabinas" OR "NISCH"))

NOT ("Ebook" OR "Kohlanta" OR "dermatite atopique" OR "atopic dermatitis" OR "eczéma atopique" OR "atopic eczema")

**Germany:** Ichthyose OR Ichthyosen OR Ichthyosis OR ichthyosiforme OR ichthyosiform OR Fischschuppenhaut OR Fischschuppen-Krankheit OR FischschuppenKrankheit OR Kollodiumbaby OR Kollodium-baby OR "collodion baby" OR trichothiodystrophy OR Trichothiodystrophie OR "Ichthyosis-Hyertrichosis" OR "Multiple Sulfatase-Defizienz" OR "Multiple sulfatase deficiency" OR "Erythrokeratodermie variabel" OR "Loricrin-Kueratodermie" OR "Loricrin Kueratodermie" OR "Loricrin-Kuratodermie" OR

Refsum-Syndrom OR Harlequin-Syndrom OR Harlequin-Ichthyose OR Harlekin-Ichthyose OR KID-Syndrom OR Netherton-Syndrom OR Sjögren-Larsson-Syndrom OR Conradi-Hünermann-Happle-Syndrom OR "Peeling-Skin Syndrom" OR Peeling-Skin-Syndrom OR IFAP-Syndrom OR Ichthyosis-Hyertrichosis-Syndrom OR MEDNIK-Syndrom OR CEDNIK-Syndrom OR Dorfman-Chanarin-Syndrom OR Ichthyose-Frühgeburt-Syndrom OR "Gaucher-Syndrom Typ 2" OR Ichthyose-Frühgeburt-Syndrom OR

(("syndrome" OR "sindrome" OR "syndrom") NEAR/3 ((Gaucher NEAR/3 "tip 2") OR "Netherton" OR "Harlequin" OR "Harlekin" OR "Sjögren Larsson" OR "Sjögren-Larsson" OR "KID" OR "SAM" OR "Refsum" OR "peeling skin" OR "peeling-skin" OR "ARCI" OR "KLICK" OR "IFAP" OR "Conradi-Hünermann-Happle" OR "Conradi Hünermann Happle" OR "TDD" OR "MEDNIK" OR "CEDNIK" OR "Neu-Laxova" OR "Neu Laxova" OR "CHIME" OR "SAM" OR "HELIX" OR "Sabinas" OR "NISCH"))

NOT ("atopische Dermatitis" OR "atopic dermatitis" OR "atopisches Ekzem" OR "atopic eczema")

**Netherlands:** Ichtyose OR ichthyosis OR ichtyosis OR ichthyosiforme OR ichthyosiform OR vissenschubziekte OR vissenhuid OR keratinisatiestoornis OR "collodion baby" OR trichothiodystrofie OR "Loricrin chueratoderma" OR "Loricrin chueratodermie" OR "Erytrokeratodermie variabel" OR "Meervoudig sulfatasedeficiëntie" OR "ichthyosis-hypotrichose-cholangitis"

OR (("syndrome" OR "sindrome" OR "syndrom" OR "syndroom") NEAR/3 ("Gaucher type 2" OR "Netherton" OR "Harlequin" OR "Harlekijn" OR "Sjögren Larsson" OR "Sjögren-Larsson" OR "KID" OR "SAM" OR "Refsum" OR "peeling skin" OR "peeling-skin" OR "ARCI" OR "KLICK" OR "IFAP" OR "Conradi-Hünermann-Happle" OR "Conradi Hünermann Happle" OR "TDD" OR "MEDNIK" OR "CEDNIK" OR "Neu-Laxova" OR "Neu Laxova" OR "CHIME" OR "SAM" OR "HELIX" OR "Sabinas" OR "NISCH"))

NOT ("atopische dermatitis" OR "atopic dermatitis" OR "atopisch eczeem" OR "atopic eczema")

**Spain:** Ictiosis OR Ichthyosis  OR ictiósico OR ictiosiforme OR ictiosiform OR "Bebé colodión" OR "collodion baby" OR "ictiosis-prematuridad" OR tricotiodistrofia OR "Quueratodermia por loricrina" OR "Eritroqueratodermia variable" OR "Deficiencia múltiple de sulfatasa" OR "ictiótica-paraplejía" OR "ictiosis-hipotricosis-colangitis" OR "ictiosis-talla" OR

(("syndrome" OR "sindrome" OR "syndrom") NEAR/3 ((Gaucher NEAR/3 "tipo 2") OR "Netherton" OR "Harlequin" OR "Arlequin" OR "Sjögren Larsson" OR "Sjögren-Larsson" OR "KID" OR "SAM" OR "Refsum" OR "peeling skin" OR "peeling-skin" OR "ARCI" OR "KLICK" OR "IFAP" OR "Conradi-Hünermann-Happle" OR "Conradi Hünermann Happle" OR "TDD" OR "MEDNIK" OR "CEDNIK" OR "Neu-Laxova" OR "Neu Laxova" OR "CHIME" OR "SAM" OR "HELIX" OR "Sabinas" OR "NISCH"))

NOT ("dermatitis atópica" OR "atopic dermatitis" OR "eczema atópico" OR "eccema atópico" OR "atopic eczema")
